# NOTIFICATION: Letters to the Editor Represent the Apogee of Peer Review: A Current Exemplar

**DOI:** 10.1113/eph.70402

**Published:** 2026-07-07

**NOTIFICATION**: T. I. Musch, K. M. Schulze, and D. C. Poole, “Letters to the Editor Represent the Apogee of Peer Review: a Current Exemplar,” *Experimental Physiology* (Early View): https://doi.org/10.1113/ep093736.

This notification is for the above article, published online on 14 March 2026 in Wiley Online Library (wileyonlinelibrary.com), and has been issued by agreement between the journal Editor‐in‐Chief, Damian Bailey; The Physiological Society; and John Wiley & Sons Ltd.

One of the authors of this article, D. C. Poole, is the Deputy Editor‐in‐Chief (USA) of *Experimental Physiology*. The manuscript underwent independent, external review before a Senior Editor made the final decision on publication. In line with journal policy, D. C. Poole was not involved in any aspect of the review process.

Notwithstanding this, the Editor‐in‐Chief and the Society consider this article to be an inappropriate use of the Editorial article type. It predominantly comprises an extensive criticism of a specific article published in another journal. It also criticises the decision of that journal to not publish additional correspondence from the authors of this editorial about that specific article.

The Editor‐in‐Chief and the Physiological Society apologise for the inappropriate use of the Editorial format and are currently reviewing the publication policies of the Society to prevent this happening in the future.

